# Occupational Exposure to Volatile Organic Compounds in Polyurethane Foam Production—Concentration, Variability and Health Risk Assessment

**DOI:** 10.3390/molecules31010145

**Published:** 2026-01-01

**Authors:** Andrzej R. Reindl, Ewa Olkowska, Jakub Pawłowski, Lidia Wolska

**Affiliations:** Department of Environmental Toxicology, Faculty of Health Sciences, Medical University of Gdansk, 80-204 Gdansk, Poland; andrzej.reindl@gumed.edu.pl (A.R.R.); jakub.pawlowski@gumed.edu.pl (J.P.); lidiawolska@gumed.edu.pl (L.W.)

**Keywords:** volatile organic compounds (VOCs), dichloromethane, polyurethane foam production, occupational exposure, health risks assessment, environmental health

## Abstract

Volatile organic compounds (VOCs) are a major occupational concern in polyurethane foam production, where exposure may impact worker health. This study identified key VOCs and evaluated their concentrations across different sections of a polyurethane manufacturing facility. Area (n = 5) air samples were collected during routine full-load production using short-duration active sampling and analyzed by thermal desorption gas chromatography–mass spectrometry (TD-GC-MS). The results revealed marked spatial variability in VOC concentrations, with the curing section showing the highest totals. Dichloromethane (DCM) constituted the dominant VOC in high-emission zones. All measured concentrations of DCM and other regulated substances remained well below European and Polish short-term exposure limits. Quantitative health risk assessment demonstrated that lifetime cancer risk values for DCM and benzene were in the 10^−6^ range, far below the regulatory threshold of concern (10^−4^). Non-carcinogenic risk indices (HQ) were generally low; however, a markedly elevated HQ was identified for 1-hexanol, 2-ethyl- in the cutting area (HQ = 5.7), indicating a potential localized non-cancer health concern. Overall, existing protective measures appear effective, but additional targeted precautions are warranted in zones with elevated emissions. Enhanced ventilation, strengthened personal protective equipment, and routine air monitoring are recommended to minimize potential health risks. Regular updates of occupational safety standards should reflect evolving toxicological evidence to ensure sustainable protection of workers in polyurethane foam production.

## 1. Introduction

Volatile organic compounds (VOCs) represent a diverse class of chemicals characterized by high vapor pressure at room temperature, which facilitates their release into the air and widespread presence in occupational environments. VOCs, including creosote derivatives, are defined by a vapor pressure of at least 0.01 kPa at 293.15 K, indicating significant volatility under standard working conditions [1]. These compounds originate from industrial processes, raw materials, and chemical reactions, making occupational exposure a relevant concern across numerous sectors such as petrochemical, printing, painting, polyurethane foam manufacturing, and services involving organic solvents [2,3,4].

Exposure to VOCs in occupational settings occurs predominantly via inhalation, although certain compounds are also capable of dermal absorption [5,6,7]. The extent of exposure is influenced by various factors, including airborne concentration, exposure duration, efficacy of local ventilation, and the use of personal protective equipment [8]. Acute exposure to VOCs may cause irritation of mucous membranes, dizziness, and central nervous system effects. Chronic exposure, particularly to carcinogenic or toxic compounds such as benzene, may result in haematological disorders, liver and kidney impairment, and increased cancer risk.

In polyurethane foam (PUR) manufacturing, toluene diisocyanate (TDI) methylene diphenyl diisocyanate (MDI) and dichloromethane (DCM) are of particular relevance due to their widespread use and toxicological profiles. TDI, a key component in PUR synthesis while MDI for rebounding processes. They are potent respiratory sensitizers capable of inducing occupational asthma and immunologic sensitization. DCM, widely employed as a blowing agent, is recognized for its neurotoxicity and potential carcinogenicity. The co-occurrence of these substances in foam production environments significantly elevates the health risks for exposed workers.

Occupational exposure to specific VOCs, particularly DCM and benzene, represents a growing concern due to their documented adverse health effects. Benzene has been classified by the International Agency for Research on Cancer [9] as a Group 1 carcinogen, indicating sufficient evidence of carcinogenicity in humans [9,10]. According to estimates by the National Institute for Occupational Safety and Health (NIOSH), approximately one million workers in the United States are potentially exposed to DCM, while occupational exposure to benzene affects around 238,000 individuals [11,12]. In the European Union, the number of workers exposed to benzene is estimated at approximately one million [13]. These figures highlight the urgent need for systematic evaluation and control of inhalation exposure to these compounds in occupational environments.

Managing VOC-related risks in the workplace requires a multifaceted strategy that includes emission source identification, continuous air monitoring, implementation of engineering controls, use of personal protective equipment, and targeted worker training [14,15,16,17]. Epidemiological studies increasingly emphasize the health burden of VOCs in industrial processes, with polyurethane foam production identified as a sector of particular concern due to its use of highly hazardous chemicals.

A key compound in polyurethane production is toluene diisocyanate (TDI), for which occupational exposure limits are extremely low. For example, ACGIH’s Threshold Limit Values (TLVs) are in the single-digit parts per billion (ppb) range—e.g., 5 ppb for an 8 h time-weighted average, with even lower values proposed in some jurisdictions. Acute reactions and sensitization can occur at concentrations as low as 1–20 ppb, indicating very narrow safety margins for airborne TDI exposure [18].

The public health relevance of such low thresholds was illustrated in Randolph County, North Carolina (1995–1997), where residents living near a polyurethane foam manufacturing facility reported widespread respiratory, dermal, and neurological symptoms associated with emissions of TDI and DCM. These concerns ultimately led to the facility’s closure and an investigation by the Agency for Toxic Substances and Disease Registry (ATSDR), which confirmed a correlation between VOCs exposure and adverse health outcomes [19]. Immunological testing detected anti-diisocyanate antibodies in some exposed individuals, suggesting the potential for long-term sensitization.

The growing body of evidence on the occupational health impacts of VOCs underscores the urgent need for robust exposure assessment frameworks and strengthened regulatory oversight. Despite this, a critical gap persists: in the polyurethane production sector, health risk assessments have been predominantly limited to isocyanates, with little to no comprehensive evaluation of the broader VOCs mixture present in real-world processes.

The novelty of the present study lies in the application of a structured health risk assessment for occupational exposure to selected VOCs, with particular focus on TDI, MDI, DCM, and benzene. The approach integrates detailed exposure characterization, toxicological evaluation, and quantitative risk estimation, all carried out under real workplace conditions. The primary objective is to provide a comprehensive evaluation of health risks associated with VOCs exposure in polyurethane foam production. In addition, the study aims to critically assess the adequacy of current legal regulations, technological safeguards, and protective measures, offering evidence-based recommendations to enhance occupational health management in this sector.

## 2. Results

### 2.1. VOC in the Workplace

**Figure 1:**
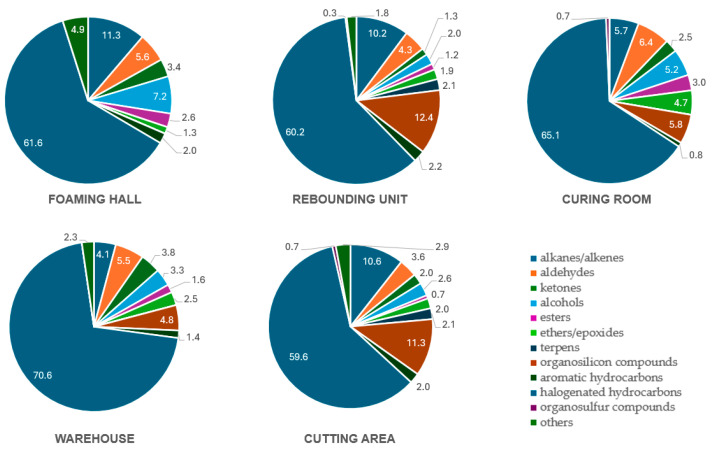
Percentage contribution of volatile organic compounds to the total VOC profile across five production zones of the polyurethane foam facility.

The composition of VOCs varied across production zones, reflecting the specific processes conducted in each area (Figure 1). Group-based analysis revealed that halogenated hydrocarbons dominated VOCs emission in all production areas, with their share ranging from 59% in the cutting room to 70% in the warehouse. Alkanes and alkenes formed the second most abundant group in several areas, reaching 11% in Foaming hall and 11% in cutting room. Organosilicon compounds were particularly prominent in rebounding unit (12%) and cutting room (11%), suggesting the presence of silicone-based materials. Lower contributions were observed for alcohols (2–7%), aldehydes (4–6%), ketones (1–4%), esters, and ethers. Aromatic hydrocarbons were present at low levels (0.8–2%) across all sections. Organosulfur compounds were detected in minor quantities (up to 0.7%) or were absent in most zones. DCM was the dominant compound in four out of five production areas, contributing 52% of the total VOCs in the foaming hall, 60% in curing room, 61% in cutting room, and peaking at 71% in the warehouse (Table 1). In the tested air samples, compounds with peak unit surface area approximately 30,000 or more and identification similarity certainty greater than 80% were catalogued. The broadest set of VOCs was identified in the cutting room (47 individual analytes).

### 2.2. Chronic Exposure Intake

Chronic exposure intake (EC) was estimated for occupational scenarios of 25 (NCR—non cancer risk) and 70 years (LCR—lifetime cancer risk) for individual VOC groups (Table 2). Halogenated hydrocarbons dominated in all production areas, there VOC groups showed lower exposure levels while terpenes and organosulfur compounds were detected only in selected areas at trace exposure levels.

### 2.3. Health Risk Assessment

Quantitative health risk assessment was performed for selected VOCs, focusing on both carcinogenic and non-carcinogenic effects (Table 3). In all production areas, lifetime cancer risk (LCR) values for DCM ranged from 1.1 × 10^−6^ (foaming hall) to 1.9 × 10^−6^ (cutting area), remaining well below the regulatory concern threshold of 1 × 10^−4^. For benzene, LCR values were even lower, from 1.2 × 10^−6^ (rebounding unit) to 2.2 × 10^−6^ (curing room). None of the assessed compounds exceeded the accepted carcinogenic risk threshold. Hazard quotient (HQ) values for non-carcinogenic effects were generally low. The highest HQ values among all VOCs were noted for 1-hexanol, 2-ethyl- in the cutting area (HQ = 5.7) and propane, 1,2-dichloro- (HQ = 0.77 in warehouse and 0.37 in cutting area), indicating potential localised non-cancer health concerns. For most other compounds, HQs were <0.1, suggesting negligible risk under current exposure conditions.

### 2.4. Remarks on Diisocyanates and Compliance with Legal Exposure Limits

Occupational exposure to 2,4-toluene diisocyanate (2,4-TDI) and methylene diphenyl diisocyanate (MDI) was not quantitatively assessed in terms of cancer or non-cancer risk due to the absence of inhalation unit risk (IUR) and reference concentration (RfC) values in the USEPA IRIS database. However, both compounds are recognized respiratory sensitizers and are classified as asthmagens. As such, they should be included in qualitative risk communication protocols and targeted preventive strategies within chemical risk management plans.

In the context of regulatory compliance, it is important to note that all measured concentrations of DCM across the examined workplace zones remain well below the legally established short-term exposure limit (STEL) of 353 × 10^3^ μg/m^3^, as defined by European Commission [20]. The highest observed DCM concentration—4.6 × 10^3^ μg/m^3^ in the curing room, represents only approximately 1.3% of the legal limit. Other measured values include 1.3 × 10^3^ μg/m^3^ in the cutting area and warehouse, 0.20 × 10^3^ μg/m^3^ in the foaming hall, and 0.011 × 10^3^ μg/m^3^ in the office section.

Although DCM is classified as a toxic substance and a suspected human carcinogen, the concentrations recorded in this study do not pose an immediate health risk under current occupational exposure limits. These findings indicate a high level of regulatory compliance, and suggest that the actual health risk under routine working conditions is minimal. Nonetheless, due to the compound’s toxicological profile and cumulative risk potential, continuous air monitoring and retention of existing exposure controls are recommended to ensure ongoing worker protection and early detection of any future exceedances.

## 3. Discussion

The results of the study conducted at the polyurethane manufacturing facility indicate significant variability in VOC concentrations across different sections of the plant. The highest concentrations were recorded in the curing section, where DCM was the dominant compound. Although the measured VOC concentrations in the facility were below the permissible limits set by European and Polish regulations, it is essential to better understand the potential health impacts of long-term worker exposure to VOCs. Air samples were collected during full production load under stable operating conditions; therefore, VOC emissions are primarily process-related and the measurements are considered representative of typical workplace exposure.

This study was based on area sampling designed to characterize typical exposure conditions within defined production zones rather than task-based or personal exposure. It is therefore possible that workers performing specific activities, such as cutting operations or handling freshly cured foam, may experience higher short-term exposures than those reflected by area-based measurements.

We identified the curing room as the zone with the highest concentration of DCM corresponding to an estimated non-cancer hazard quotient (HQ) of 0.66 and a lifetime cancer risk (LCR) of 1.7 × 10^−6^. These values, while below regulatory thresholds, suggest moderate long-term risk. 

The findings are consistent with the literature on VOC emissions and exposure in industrial polyurethane foam production processes. The predominance of DCM in VOC emissions confirms previous scientific reports indicating the widespread use of this compound as a solvent and foaming agent in PUR foam production. Studies such as the report by Hillier et al. [2] have shown that these processes generate significant amounts of VOCs, including DCM, which can pose a considerable health risk to workers.

Our results confirm that the proportion of DCM in total VOC emissions was highest in the warehouse (70%), curing (65%), and foaming hall (61%) zones, matching elevated levels of DCM in confined processing environments. This findings also support the relevance of observed neurotoxic and cardiotoxic effects following DCM exposure [21].

Our findings regarding VOC emission profiles in polyurethane foam production are consistent with previous research on VOC emissions from polyurethane materials. Chamber studies on polyurethane foams and related products have demonstrated that fresh urethane materials can emit a range of VOCs, including aliphatic and aromatic hydrocarbons, alcohols, and chlorinated compounds, with emission rates and profiles depending on material composition and environmental conditions. For example, investigations of VOC emissions from flexible polyurethane foam products showed the presence of various VOCs released from the material surface, and overall VOC emissions were found to differ substantially among samples without exceeding regulatory thresholds under controlled conditions [22]. While specific data on dichloromethane dominance in industrial settings are limited, the observed variability in VOC profiles is in line with chamber studies of polyurethane foam emissions, supporting the notion that process-related emissions can vary by compound and production stage. This aligns with the current study’s identification of dichloromethane as a prominent VOCs in high-emission zones such as the curing section, reflecting process-specific contributions to workplace air quality.

Epidemiological studies, such as those conducted in Randolph County, North Carolina, indicate that exposure to DCM and TDI led to various health symptoms, including headaches, respiratory irritation, coughing, and skin problems among residents and workers in nearby PUR manufacturing plants [23,24,25,26,27]. While the VOC concentrations measured in our facility were considerably lower, the health effects reported in the literature suggest that cumulative or prolonged exposure, even at moderate levels, warrants precautionary oversight.

Importantly, none of the measured DCM concentrations in this study exceeded the short-term exposure limit (STEL) of 353 × 10^3^ μg/m^3^ set by the European Commission Directive 2017/164 and Polish national regulation (Dz. U. 2018 poz. 1286). The maximum recorded DCM level in the curing room represented less than 1.5% of the legal STEL. This supports regulatory compliance, yet the HQ > 1 in the cutting zone indicates that STEL compliance alone may not fully reflect chronic health risks—an observation echoed in [8], where permissible exposure limits (PELs) differ from health-protective reference values. Although the regulatory comparison focused primarily on STELs due to the episodic nature of VOC emissions, the measured concentrations were also substantially below available time-weighted average (TWA) limits, indicating a low likelihood of adverse effects associated with chronic exposure.

The notably high HQ and cancer risk values in the cutting and curing sections suggest that current control measures may not fully eliminate long-term exposure risks. The health risk assessment presented in Table 3 demonstrates that lifetime cancer risks (LCR) for DCM across all production areas and for benzene remain well below the commonly accepted threshold. These findings are consistent with previous industrial VOC assessments, which generally report low carcinogenic risk levels in occupational settings when exposure levels are within established guidelines [28].

However, certain non-carcinogenic hazard quotients (HQ) merit attention. While DCM HQs stayed below 1.0 (e.g., up to 0.66 in the curing room), compounds like 2-ethyl-1-hexanol (2EH), (HQ = 5.7 in the cutting area) indicating localized non-carcinogenic risk. 2EH is a known VOC associated with industrial materials and plasticizers, and may be present in various auxiliary materials used in polyurethane processing. Elevated presence of 2EH in the cutting area may reflect emissions from polyurethane materials and auxiliary products subjected to mechanical stress during cutting operations. In the context of the facility studied, the mechanical processing of polyurethane foam (cutting) likely contributed to episodic releases of 2EH. Mechanical stress and abrasion of foam and associated products can enhance the desorption or volatilization of previously absorbed or surface-bound VOCs. This compound has been reported in other indoor air studies as a volatile emission product from complex material sources [29]. Similar findings have been reported in workplace studies where specific VOCs, though often overlooked, pose health risks due to elevated chronic exposure or mixture effects [30].

Empirical comparisons with industries involving polyurethane foam echo comparable concerns. The Danish EPA’s survey of consumer PU foam items revealed concerning emissions, such as dimethylformamide (DMF), capable of inducing respiratory irritation and reproductive toxicity with prolonged exposure [31]. This underscores the potential for additive-based or off-gassing VOCs to warrant risk mitigation even when regulatory thresholds are not breached.

Moreover, recent comprehensive reviews highlight persistent challenges in managing VOC emissions, particularly halogenated and aromatic hydrocarbons, in foam production settings, advocating improved process controls and ventilation strategies to reduce cumulative exposure [32].

In a regulatory context, the values we observed remain below occupational exposure limits (OELs) such as IOELVs set by EU regulations for DCM and benzene, which typically range between 0.2 and 0.66 × 10^3^ μg/m^3^ [33,34]. Nevertheless, the presence of elevated HQs for certain compounds supports recommendations for site-specific monitoring, task-based exposure assessment, and implementation of control measures, such as improved ventilation, enclosed operations, personal protective equipment, and substitution where feasible, aligned with best practices promoted by occupational hygiene guidelines.

The role of diisocyanates (2,4-TDI and MDI), although not quantitatively assessed due to the absence of IUR/RfC values in IRIS, remains critical. These compounds are known asthmagens and respiratory sensitizers. Their measured concentrations, although low, were observed in foam-generating and rebonding areas. This supports the findings of NIOSH [35], which highlights the sensitizing potential of diisocyanates even at sub-threshold levels. Although diisocyanates such as TDI and MDI were not included in the quantitative risk assessment due to the lack of inhalation unit risk and reference concentration values, their presence remains critical from an occupational health perspective. As potent respiratory sensitizers and asthmagens, these compounds may contribute to overall health risks at low exposure levels, underscoring the importance of preventive measures, engineering controls, and medical surveillance beyond VOCs-based risk metrics.

Overall, our results reinforce the need for enhanced air monitoring, task-specific PPE, and potentially upgrading ventilation infrastructure in high-risk areas. Integrating quantitative risk assessments with process-specific control strategies is essential for proactively protecting worker health. The use of personal protective equipment, particularly respiratory protection during high-emission tasks, may substantially reduce actual inhalation exposure and should be considered an important complementary control measure alongside ventilation and process design based on best available techniques requirements.

## 4. Materials and Methods

### 4.1. Facility Description and Production Characteristics

The study was conducted at an industrial facility specializing in the manufacture and processing of flexible polyurethane foams, with a particular focus on occupational exposure to VOCs and the associated health risks. The facility operates continuous processing lines employing MaxFoam^®^ technology, which involves large-scale, high-throughput production and is representative of contemporary industrial practices in this sector.

The production workflow encompasses the following stages: precise metering and mixing of polyol and isocyanate components, the foaming reaction, polymer stabilization, thermal curing, and mechanical conversion of foam blocks into finished formats. Post-production, scrap material is recovered and repurposed into rebounded foam, aligning with principles of sustainable manufacturing and waste minimization.

In accordance with the SEVESO III Directive [20], the facility is classified as a high-risk establishment due to the on-site handling and storage of hazardous substances, notably toluene diisocyanate (TDI). This classification obliges the company to implement rigorous risk management protocols, including continuous safety monitoring, periodic hazard identification and assessment, and comprehensive emergency preparedness plans to prevent or mitigate chemical incidents such as leaks, explosions, or uncontrolled emissions.

### 4.2. Air Sampling Protocol

Sampling of airborne VOCs was carried out on 16 May 2024, at six designated locations within the facility to capture spatial variability in exposure across production and non-production zones. The production-related samples were collected in distinct building units, representing separate operational areas: Foaming Hall, Rebounding Unit, Curing Room, Warehouse, and Cutting Area. This approach ensured that measurements reflected the specific processes and emission profiles characteristic of each production zone. Air samples were collected using Tenax TA sorbent tubes (1/4″ × 90 mm, filled with 130 mg of sorbent; Shimadzu Corp., Kyoto, Japan), which were preconditioned at 280 °C under a 2.5 bar flow of high-purity helium (99.9999%) to eliminate potential contaminants. The use of Tenax TA sorbent ensure methodological consistency for relatively broad group of VOCs and across all sampling locations, where storage and transport of samples to laboratory was needed [36]. Prior to sampling, each tube was spiked with 48.1 ng of 1-bromo-4-fluorobenzene, used as an internal standard (IS) due to its chemical stability, absence in workplace air and suitability in VOCs analysis while keeping costs contained [37,38,39].

Air was drawn through the tubes using a manual sampling pump at the following locations: foaming hall, rebounding unit, curing room, warehouse and cutting area. Sampling volumes ranged from 100 mL to 500 mL dependence on the locations. The applied sampling volumes (100–500 mL) were selected to balance analytical sensitivity with sorbent capacity and to minimize the risk of analyte breakthrough. Sampling was performed by drawing a defined air volume through the sorbent using a calibrated manual device, corresponding to an approximate flow rate of 100 mL·min^−1^ and a total sampling duration of 1–5 min. Immediately after collection, tubes were sealed and stored at ambient temperature for transport to the analytical laboratory. Blank control samples were collected concurrently to verify background contamination.

### 4.3. Analytical Procedure for VOC Identification

VOC determination was performed within four hours post-sampling using a thermal desorption-gas chromatography-mass spectrometry (TD-GC-MS) system (Shimadzu Corp., Kyoto, Japan). Tubes were thermally desorbed at 250 °C in a helium stream (1.0 mL/min). Compounds were separated on a Zebron™ ZB-624 capillary column (60 m × 0.32 mm, 1.8 μm film thickness) (Phenomenex, Torrance, CA, USA) and detected using mass spectrometry in SCAN mode (*m*/*z* 30–450).

Chromatographic data were analyzed using LabSolutions software (Shimadzu Corp., Kyoto, Japan), which identified VOCs based on retention times and spectral patterns. Automated spectral matching with the NIST library enabled high-confidence identification. Quantification was achieved via peak area integration relative to the internal standard, and results were expressed in μg/m^3^. Changes in IS response were evaluated by systematically monitoring its signal between analytical runs (the coefficient of variation obtained below 5%).

### 4.4. Supplementary Exposure Assessment and Verification

Occupational exposure concentrations for TDI (6.3 ± 1.1 μg∙m^−3^) and MDI (1.1 ± 0.3 μg∙m^−3^) were determined from workplace monitoring data. Reported values represent five-year arithmetic means derived from systematic measurements conducted at designated workstations. Although VOCs sampling in this study was performed as a single campaign, long-term monitoring of other regulated occupational agents has consistently confirmed stable production conditions and effective process control. This supports the assumption that the measured VOCs concentrations are representative of routine exposure levels under normal operating conditions.

To ensure a comprehensive evaluation, additional exposure data were obtained from an accredited external laboratory in line with national occupational hygiene regulations. These supplementary measurements covered indoor air quality in critical production zones—including primary foaming, secondary processing, curing, cutting, packaging, and warehousing—as well as office areas, thereby accounting for all potentially exposed personnel.

The integration of these multi-source datasets enabled verification and refinement of VOCs exposure estimates across distinct operational zones. Combined with direct analytical results, this approach provided a spatially resolved VOCs distribution profile, forming a solid basis for quantitative health risk assessment and the development of targeted intervention strategies.

### 4.5. Exposure and Health Risk Assessment

Inhalation exposure to VOCs among industrial workers was assessed using a deterministic approach based on the United States Environmental Protection Agency [35] risk assessment guidelines and other further studies [40,41,42]. The exposure concentration (*EC*) for non-cancer risk (*EC_NCR_*) and for lifetime cancer risk (*EC_LCR_*) in air was estimated using the following equation:

$$EC=\frac{CA\cdot ET\cdot EF\cdot ED}{AT}$$

*where*

− *CA is the concentration of VOCs in inhaled air (µg/m^3^),*
− *ET is the exposure time in hours per day (assumed as 8 h/day or longer depending on individual worker data),*
− *EF is the exposure frequency (5 days/week × 50 weeks/year = 250 days/year),*
− *ED is the exposure duration (25 years),*
− *AT is the averaging time, calculated as:*
  ○ *EC_NCR_—non-cancer risk estimation: 219,150 h for occupational exposure (25 years)*
  ○ *EC_LCR_—lifetime cancer risk assessment: 613,200 h for (70 years × 365 days/year × 24 h/day).*

Lifetime cancer risk (*LCR*) characterization was performed by comparing the *EC_(LCR_*_)_ to the inhalation unit risk (*IUR*) value, using the formula:

$$LCR=IUR\cdot {EC}_{\mathrm{LCR}}$$

The *IUR* for VOCs was based on USEPA IRIS data. A cancer risk value: >1 × 10^−6^ is considered unacceptable, whilst < 1 × 10^−6^ is considered acceptable, in accordance with international standards.

Non-carcinogenic risk was evaluated using the Hazard Quotient (*HQ*), defined as:

$$HQ=\frac{EC}{RfC}$$

where the reference concentration (*RfC*) for VOCs for chronic inhalation exposure, adopted according to USEPA IRIS. *HQ* > 1 indicates an unacceptable non-cancer risk, whereas *HQ* < 1 suggests an acceptable risk.

## 5. Conclusions

This study comprehensively evaluated occupational exposure to volatile organic compounds in a polyurethane foam production facility. DCM was identified as the predominant compound, with the highest concentrations recorded in the curing room, cutting areas and rebounding unit. Nevertheless, all measured levels remained well below established occupational exposure limits, indicating that current workplace conditions are effectively controlled.

Quantitative health risk assessment confirmed that both carcinogenic and non-carcinogenic risks associated with VOC exposure were negligible across all operational areas. Even in the curing and cutting zones, where concentrations peaked, the estimated values of lifetime cancer risk and hazard quotients did not exceed levels of regulatory concern. These results suggest that existing engineering controls and protective measures provide adequate worker protection under current operating conditions.

Unlike many previous studies focusing narrowly on isocyanates, the present work extends the scope of analysis to a wider spectrum of VOCs. This broader evaluation demonstrates that, although trace levels of multiple compounds are detectable, their combined health impact in polyurethane foam production appears minimal.

## Figures and Tables

**Table 1:** VOC concentration in production zones with the emphasis on dichloromethane.

Production Zone	No. of VOCs	ΣVOCs	Dichloromethane	
	[-]	Concentration [µg/m^3^]	Concentration [µg/m^3^]	Contribution [%]
Foaming hall	38	1670	1026	61
Rebounding unit	42	2596	1556	60
Curing room	37	7119	4631	65
Warehouse	43	1845	1287	70
Cutting area	47	2445	1446	59

**Table 2:** Measured concentrations and estimated chronic exposure intake (EC) of selected VOCs in operational areas of a polyurethane foam facility calculated for occupational exposure (LCNCR) and for cancer risk assessment (ECLCR) in ug/m^3^.

Group of VOCs	Foaming Hall		Rebounding Unit		Curing Room		Warehouse		Cutting Area	
	EC_NCR_	EC_LCR_	EC_NCR_	EC_LCR_	EC_NCR_	EC_LCR_	EC_NCR_	EC_LCR_	EC_NCR_	EC_LCR_
Alcohols	0.021	7.3	0.029	3.2	0.043	15	0.012	4.3	0.014	4.8
Aldehydes	0.24	5.0	0.39	6.8	0.71	19	0.33	6.2	0.45	6.7
Alkanes/Alkenes	0.032	11	0.045	16	0.046	13	0.017	5.9	0.056	19
Ketones	0.0098	3.5	0.0059	2.1	0.0202	7.2	0.014	5.0	0.011	3.8
Halogenated hydrocarbons	0.18	63	0.27	95	0.53	188	0.26	93	0.31	111
Aromatic hydrocarbons	0.057	2.0	0.0099	3.6	0.0069	2.5	0.0052	1.9	0.011	3.8
Esters	0.076	2.7	0.012	4.4	0.025	8.8	0.0058	2.0	0.0034	1.2
Terpens	NE	NE	0.0095	3.4	NE	NE	NE	NE	0.011	4.0
Ethers/epoxides	0.038	1.4	0.0085	3.5	0.038	14	0.0085	3.0	0.0084	3.7
Organosulfur compounds	NE	NE	0.0012	0.42	0.0054	1.9	NE	NE	0.0036	1.3
Organosilicon compounds	NE	NE	0.018	6.3	0.036	17	0.013	6.3	0.019	6.9
Others	NE	NE	0.014	5.0	0.0011	0.41	0.0085	3.0	0.015	5.4

*NE—not estimated*.

**Table 3:** Quantitative health risk assessment of VOC exposure based on inhalation unit risk (LCR—cancer risk) and hazard quotient methodologies (HQ—non cancer risk).

VOCs Assessed	Foaming Hall		Rebounding Unit		Curing Room		Warehouse		Cutting Area	
	HQ	LCR	HQ	LCR	HQ	LCR	HQ	LCR	HQ	LCR
Methyl Alcohol	0.0053	-	0.0018	-	0.011	-	0.0035	-	0.0020	-
Pentane	0.0012	-	0.0071	-	-	-	-	-	0.0064	-
Dichloromethane	0.29	1.1 × 10^−6^	0.44	1.6 × 10^−6^	0.66	1.7 × 10^−6^	0.43	1.6 × 10^−6^	0.52	1.9 × 10^−6^
Pentane, 3-methyl-	-	-	0.028	-	-	-	-	-	-	-
2-Butanone	-	-	0.00044	-	-	-	-	-	-	-
n-Hexane	0.015	-	-	-	-	-	0.0017	-	0.017	-
2-Butanone	0.00033	-	-	-	-	-	-	-	0.00033	-
Benzene	0.053	1.3 × 10^−6^	0.049	1.2 × 10^−6^	0.17	2.2 × 10^−6^	0.054	1.7 × 10^−6^	0.052	1.2 × 10^−6^
Propane, 1,2-dichloro-	-	-	-	-	-	-	0.77	-	0.37	-
Toluene	0.00072	-	0.0014	-	-	-		-	0.0015	-
o-Xylene	0.0051	-	-	-	-	-	-	-	-	-
Benzene, 1,3-dimethyl-	-	-	-	-	-	-	-	-	0.014	-
Octanal	-	-	-	-	-	-	-	-	0.034	-
Ethanol, 2-butoxy-	0.0027	-	-	-	-	-	-	-	-	-
1-Hexanol, 2-ethyl-	-	-	-	-	-	-	-	-	5.7	-
Benzaldehyde	0.029	-	0.031	-	0.061	-	0.022	-	-	-

## Data Availability

The original contributions presented in this study are included in the article. Further inquiries can be directed to the corresponding author.
